# An integrated workflow for phosphopeptide identification in natural killer cells (NK-92MI) and their targets (MDA-MB-231) during immunological synapse formation

**DOI:** 10.1016/j.xpro.2023.102104

**Published:** 2023-02-10

**Authors:** Daniel Perez-Hernandez, Liza Filali, Clement Thomas, Gunnar Dittmar

**Affiliations:** 1 Department of Infection and Immunity, Luxembourg Institute of Health, Strassen, Luxembourg; 2 Department of Cancer Research, Luxembourg Institute of Health, Strassen, Luxembourg; 3 Department of Life Sciences and Medicine, University of Luxembourg, Belval, Luxembourg

**Keywords:** Cell Biology, Flow Cytometry/Mass Cytometry, Immunology, Molecular/Chemical Probes, Protein Biochemistry, Proteomics, Mass Spectrometry

## Abstract

Here, we present a protocol to identify and quantify phosphopeptides during the dynamic formation of an immunological synapse. We describe steps for mixing isotope-labeled immune and target cells, the stabilization of cell-to-cell conjugates by cross-linking, and their isolation by fluorescence-activated cell sorting. We detail the isolation of phosphopeptides by phosphopeptide enrichment and their subsequent measurement by mass spectrometry. Finally, we describe the analysis of the resulting data to separate cell-specific phosphopeptides using the isotope label and label-free quantification.

## Highlights

• Isolation of NK cells forming an immune synapse
• Cell-type-specific phosphoproteomics
• SILAC-based quantification of the phosphopeptides
• Mass-spectrometry-based quantification of the phosphopeptides

## Abstract (editor highlights)

Publisher’s note: Undertaking any experimental protocol requires adherence to local institutional guidelines for laboratory safety and ethics.

## Graphical abstract

**Figure:**
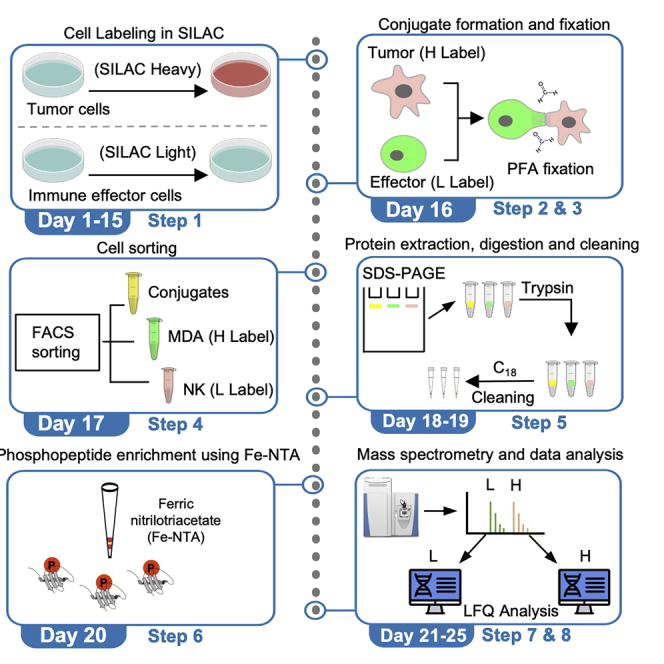

## Before you begin

Rapid killing of diseased cells, such as cancer cells, by cytotoxic lymphocytes relies on a highly specialized cell-to-cell conjugation termed the immunological synapse. Formation and activity of the immunological synapse involve multiple sequential steps, including but not limited to, recognition of and adhesion to the target cell, integration of signals at the target cell surface driving activation of effector functions, polarization of the lytic machinery toward the target cell and secretion of effector molecules, such as granzyme B and perforin, into the synaptic cleft.^1^^,^^2^^,^^3^

Cancer cells have evolved defense responses to evade lymphocyte cytotoxicity, such as rapid polarization of their actin cytoskeleton and vesicles toward the immunological synapse.^4^^,^^5^^,^^6^ Characterization of the signaling pathways controlling the key cellular processes at both pre- and post-synaptic sides of the immunological synapse is essential to develop novel strategies aimed at improving cytotoxic lymphocyte immune response.

In this protocol we describe the steps for the formation of immunological synapses between natural killer (NK) cells and target cancer cells, the stabilization and isolation of cell-to-cell conjugates, the enrichment and analysis of phospho-peptides by mass spectrometry, and the specific data analysis allowing to unravel phospho-signaling in each cell type. Although the protocol is detailed it is required that the experimenter is familiar with the main techniques used in the protocol, mass spectrometry of peptides, the analysis of proteomics data, and FACS sorting. The protocol can be used with less than 25 μg starting material because of the high efficiency of the preparation and the accuracy of the analysis.

### Protocol overview

**Figure 1:**
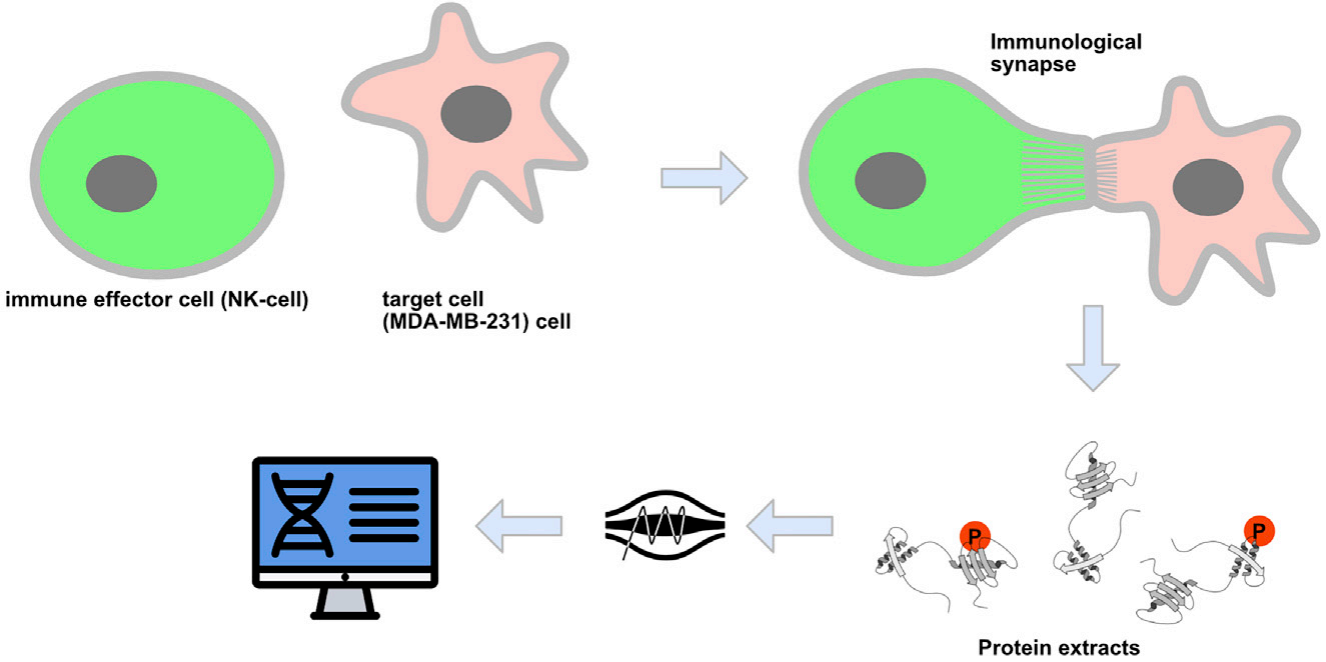
Overview of the experimental setup SILAC-labeled NK92-MI cells are incubated with MDA-MB-231 (target cells) and establish an immunological synapse. NK92-MI -target cell conjugates are isolated based on fluorescence (red + green), and the proteins from these conjugates are extracted. After enrichment of the phosphopeptides the samples are analyzed by mass spectrometry and the resulting data is processed in silico.

Although exemplified for the analysis of the lytic synapse between NK cells and tumor cells, our protocol could, in principle, apply to other types of immunological synapses, such as those involving T cells, dendritic cells, or B cells.^1^^,^^7^^,^^8^^,^^9^ The main principle of this protocol is that the two populations of interacting cells are labeled with different SILAC amino acids,^10^^,^^11^ allowing to trace back the origin of the phosphopeptides identified by mass spectrometry. Light-labeled (L) NK cells and heavy-labeled (H) breast cancer MDA-MB-231 cells are cocultured for a short period of time to allow immunological synapse formation. Cell-to-cell conjugates are then stabilized by chemical cross-linking and isolated using FACS sorting. Proteins are extracted and separated on an SDS-PAGE, and proteins are digested *in situ*. Phosphopeptides are enriched by Fe-chelate-chromatography and analyzed by mass spectrometry. Recorded spectra are analyzed using the MaxQuant software package,^12^^,^^13^ followed by bioinformatic data analysis (Figure 1).

#### Prepare buffers and reagents

**Timing: 0.5–2 h**

1. Prepare buffers following the recipes in the *materials and equipment.*
2. All reagents can be stored at room temperature for 2 months, except the ones indicated by the manufacturer:
  a. Trypsin should be prepared fresh at 0.25 μg/μL in 50 mM ammonium bicarbonate buffer.
  b. Reduction buffer and Alkylation buffer should be prepared fresh.

***Note:*** Alkylation buffer should be kept in the dark.

***Note:*** The protocol below describes the sample preparation for approximately 25 samples. For a larger number of samples, the volumes need to be adjusted.

## Key resources table

**Table:** 

REAGENT or RESOURCE	SOURCE	IDENTIFIER
**Antibodies**		

PE/Cyanine7 anti-human CD56 (NCAM) antibody	BioLegend	Cat# 318318, RRID: AB_604107

**Chemicals, peptides, and recombinant proteins**		

H_2_O, LC-MS grade	Supelco	7732-18-5
Precision Plus Protein™ Dual Color Standards	Bio-Rad	1610374
10× Tris/Glycine/SDS	Bio-Rad	1610732
2× Laemmli sample buffer	Bio-Rad	1610737
InstantBlue	Abcam	ISB1L-1L
Dithiothreitol (DTT)	Fisher Scientific	3483-12-3
2-Cloroacetamide (CAA)	Aldrich	22790-250G-F
Trypsin	Promega	V5111
Formic acid LC-MS grade	ThermoScientific	85178
Ammonium bicarbonate	Fluka	09830-500G
Acetonitrile	Supelco	EM-AX0145P-6
Urea	Sigma-Aldrich	U5128
Tris-HCl	Merck	1185-53-1
NaCl	Merck	7647-14-5
EDTA	Merck	60-00-4
16% formaldehyde, methanol-free (PFA)	Thermo Fisher Scientific	Cat #28908
Methanol HPLC grade	Sigma-Aldrich	900688
RPMI 1640 Medium for SILAC	Thermo Fisher Scientific	Cat# 88365
DMEM:F-12 for SILAC	Thermo Fisher Scientific	Cat# 88370
Fetal bovine serum	Sigma-Aldrich	Cat# F0392
L-Arginine monohydrochloride	Sigma-Aldrich	Cat# A6969
L-Arginine · HCl (^13^C_6_, 99%; ^15^N_4_, 99%)	Cambridge Isotope Laboratories	Cat# CNLM-539-H-0.05
L-Lysine monohydrochloride	Sigma-Aldrich	Cat# L8662
L-Lysine · 2HCl (^13^C_6_, 99%; ^15^N_2_, 99%)	Cambridge Isotope Laboratories	Cat# CNLM-291-H-0.05
L-Leucine	Sigma-Aldrich	Cat# L8912
Penicillin/Streptomycin	Lonza	Cat# DE17-602E
Puromycin dihydrochloride	Santa Cruz	Cat# CAS 58-58-2
Dulbecco’s phosphate-buffered saline (DPBS), 1**×**, no calcium, no magnesium	Thermo Fisher Scientific	Cat# 10010015
AutoMACS Running Buffer	Miltenyi Biotec	Cat #130-091-221
Dulbecco’s phosphate-buffered saline (DPBS), 1**×** with Ca^2+^ & Mg^2+^	Thermo Fisher Scientific	Cat# 14040-091
PBS with Mg^2+^ and Ca^2+^ (PBS/Mg/Ca)	Thermo Fisher Scientific	Cat# 14040133
Trypsin-EDTA	Thermo Fisher Scientific	Cat# 25300062

**Critical commercial assays**		

MycoAlert Mycoplasma Detection kit	Lonza	Cat# LT07-318
Live-or-Dye NucFix Red Staining kit	Biotium	Cat# 32010
High-Select Fe-NTA Phosphopeptide Enrichment Kit	Thermo Fisher Scientific	A32992
Pierce™ BCA Protein Assay Kit	Thermo Fisher Scientific	Cat# 23225
Trypan Blue Solution 0.4%	Thermo Fisher Scientific	Cat# 15250-061

**Deposited data**		

Proteomics dataset	MASSIVE proteomeXchange	ftp://massive.ucsd.edu/MSV000090645/

**Experimental models: Cell lines**		

NK-92MI	ATCC	Cat# CRL-2408, RRID:CVCL_3755
MDA-MB-231	ATCC	Cat# HTB-26, RRID:CVCL_0062

**Software and algorithms**		

FlowJo v10.8.1	BD Bioscience https://www.flowjo.com/solutions/flowjo/downloads	
MaxQuant	https://maxquant.org	version 1.6.17.0
Perseus	https://maxquant.org	Version 1.6.15.0
R	http://r-project.org	Version 4.1.3

**Other**		

Thermomixer	Eppendorf	5355 000.011
SDS-PAGE Vertical Electrophoresis Cell	Bio-Rad	1658005
Microcentrifuge tubes, 2 mL	Eppendorf	88379
Vortex	Heidolph	541-10000-00
Centrifuge	Beckman Coulter	A46472
Power basic power supply	Bio-Rad	1645050
12% Mini-PROTEAN® TGX™ Precast Protein Gels	Bio-Rad	4561045
Razor blade		
pH indicator paper	Merck	1.09565.0001
0.2 mL Skirted 96-well Robotic Plate	Thermo Scientific	AB-1300
Silicone sealing mat. For 96 well PCR plates	Nerbeplus	04-090-0000
Acclaim PepMap RSLC 75μm × 2 cm nanoViper	Thermo Fisher	PN164534
Acclaim PepMap RSLC 75μm × 15 cm nanoViper	Thermo Fisher	PN164535
Q Exactive™ HF hybrid quadrupole-Orbitrap mass spectrometer	Thermo Fisher	P/N 1372090
Dionex Ultimate 3000 HPLC NCS-3500RS	Thermo Fisher	5041.0010
Autosampler WPS-3000TPL RS	Thermo Fisher	Autosampler WPS-3000TPL RS
GPS200 pipette tip	Eppendorf	0030000889
Octadecyl (C18)-bonded silica	3M	2215
Tube with round bottom for culture 5 mL	Falcon	Cat# 352054
Protein LoBind tubes, 5 mL, with snap cap	Eppendorf	Cat# 0030108302
Tube, 15 mL, pp, 17/120 mm, conical bottom	Greiner	Cat# 188271
Cell culture flask, 550 mL, 175cm^2^, PS	Greiner	Cat# 660175
Cell culture flask, 250 mL, 75cm^2^, PS	Greiner	Cat# 658175
BD FACS Aria flow cytometer	Becton Dickinson Biosciences

***Note:*** MDA-MB-231 were purchased from ATCC and subsequently genetically modified to stably express a fluorescent actin reporter for our specific research interest (Al Absi et al.^4^).

## Materials and equipment

### Buffers resource table

**Table:** NK-92MI medium

Reagent	Final concentration	Amount
RPMI 1640 medium for SILAC		400 mL
FBS dialyzed by ultracentrifugation 0.15 M NaCl	20%	100 mL
L-Arginine monohydrochloride	1% 100 μg/mL	5 mL
L-Lysine monohydrochloride	1% 100 μg/mL	5 mL
L-Leucine	1% 100 μg/mL	5 mL
Penicillin/Streptomycin	1%	5 mL
**Total**	**N/A**	**520 mL**

***Note:*** All reagents are mixed and stored at 4°C for up to 1 month, except for amino acids that are stored at −20°C and added just before using the medium.

**Table:** MDA-MB-231 medium

Reagent	Final concentration	Amount
DMEM:F-12 for SILAC		450 mL
FBS dialyzed by ultracentrifugation 0.15 M NaCl	10%	50 mL
L-Arginine · 2HCl (^13^C_6_, 99%; ^15^N_4_, 99%)	1% 100 μg/mL	5 mL
L-Lysine · 2HCl (^13^C_6_, 99%; ^15^N_2_, 99%)	1% 100 μg/mL	5 mL
L-Leucine	1% 100 μg/mL	5 mL
Penicillin/Streptomycin	1%	5 mL
Puromycin dihydrochloride	0.5 μg/mL	250 μL
**Total**	**N/A**	**520.25 mL**

***Note:*** All reagents are mixed and stored at 4°C for up to 1 month except for amino acids that are stored at −20°C and added just before using the medium.

**Table:** Lysis Buffer

Reagent	Final concentration	Amount
Urea	8 M	9.6 g solid urea in 9.6 mL of H_2_0
NaCl	75 mM	1.5 mL 1 M NaCl
Tris-HCl pH 8.0	50 mM	1 mL 1 M Tris HCl pH 8.0
EDTA	1 mM	40 μL of 500 mM EDTA
H_2_O, LC-MS grade	N/A	7.86 mL
**Total**	**N/A**	**20 mL**

• Dilution Buffer: 50 mM Tris-HCl pH 8.0.
• Washing Buffer A: dissolve 59.3 mg ammonium bicarbonate in 15 mL MS-grade water, pH 8.5.
• Washing Buffer B: take 5 mL of acetonitrile and 39.5 mg ammonium bicarbonate and fill up to 10 mL with MS-grade water, pH 8.5.
• Reduction buffer: Add 19.8 mg ammonium bicarbonate and 77.1 mg DTT and fill up to 5 mL with MS-grade water, pH 8.5.
• Alkylation buffer: Add 19.8 mg ammonium bicarbonate and 46.8 chloroacetamide and fill up to 5 mL with MS-grade water, pH 8.5.
• Ammonium bicarbonate buffer: Dissolve 39.5 g ammonium bicarbonate in 100 mL MS-grade water, pH 8.5.
• Digestion Buffer: Add 1 μg Trypsin in 150 μL ammonium bicarbonate buffer (150 μL per sample), pH 8.5.
• Mobile phase A: Dilute 100 μL formic acid in 100 mL MS-grade water (0.1%).
• Mobile phase B: Dilute 100 μL formic acid in 100 mL HPLC-grade acetonitrile.
• ST buffer A: 2 mL 100 mL HPLC-grade methanol.
• ST buffer B: Take 5 μL formic acid, 250 μL acetonitrile and fill up to 5 mL with MS-grade water.
• ST buffer C: Take 5 μL formic acid, 4 mL acetonitrile and fill up to 5 mL with MS-grade water.
• Stop buffer: Take 20 μL formic acid and fill up to 2 mL with MS-grade water.
• Fixation buffer: Dissolve 200 mg of paraformaldehyde in 10 mL double distilled water.

***Note:*** All reagents are mixed and stored at 4°C for up to 1 month.

### Mass spectrometry parameters

This protocol is specifically set up for phosphopeptide analysis in low concentration. The conditions are optimized for Ultimate 3000 UHPLC system (DIONEX) coupled to Q Exactive HF mass spectrometer (ThermoFisher Scientific), using the Nanospray Flex ion source at 275°C (ThermoFisher Scientific). The MS was calibrated following the manufacturer instructions. The MS was operated in data-dependent mode with the following parameters:

**Table:** 

**General**	
Runtime	0–149 min
Polarity	Positive
Default Charge State	2
**Full MS**	
Microscans	1
Resolution	60000
AGC target	3e6
Maximum IT	120 MS
Number of scan ranges	1
Scan range	375–1,700 m/z
Spectrum data type	Profile
**dd-MS**^**2**^	
Microscans	1
Resolution	60000
AGC target	2e5
Maximum IT	90 ms
Loop count	7
MSX count	1
Isolation Window	1.6 m/z
Isolation offset	0.0 m/z
Fixed first mass	100.0 m/z
NCE	28
Spectrum data type	Profile
**dd Settings**	
Maximum AGC target	4.50e3
Charge exclusion	Unassigned, 1, 6–8, >8
Exclude isotopes	On
Dynamic exclusion	20.0 s

### UHPLC parameters

The UHPLC setup was injecting in two-column mode: the loading pump injects the sample in the loop in “microliter pickup mode”, and sample is transferred to the Acclaim PepMap RSLC 75μm × 2 cm nanoViper (pepTrap) for 8 min at 5 μL/min. After 8 min, the column-oven valve switches to put both pepTrap and the Acclaim PepMap RSLC 75μm × 15 cm nanoViper online to start the gradient. The parameters are described below:

**Table:** 

Time (min)	Flow (μL/min)	% Mobile phase B
0	0.300	2
8	0.300	2
134	0.300	35
134.1	0.300	90
138	0.300	90
138.1	0.300	2
149	0.300	2

**CRITICAL:** Formic acid is corrosive and irritant. Handle inside a chemical hood and use appropriate safety gear.

**CRITICAL:** Methanol is a flammable liquid and poisonous by inhalation, prepare aliquots and handle inside a chemical hood.

***Alternatives:*** Other LC-MS/MS setups can be used for the MS analysis, as long as they can analyze SILAC-labeled samples.^10^^,^^14^ The conditions must be adjusted according to the instrumentation, and the final concentrations and volumes of the samples have to be adjusted. Other SDS-PAGE systems can be used for sample cleaning and fractionation, but it is important to keep the protein amount at 25 μg for further phosphopeptide enrichment and mass spectrometry analysis.

## Step-by-step method details

### Production of SILAC-labeled NK-target cell conjugates

#### Cell labeling in SILAC medium

**Timing: 3 weeks (for steps 1 to 2)**

This section describes the labeling of the cells in SILAC media. NK-92MI cells are cultured in light-labeled and the MDA-MB-231 cells in heavy-labeled SILAC media.^10^ Duration of cell culture in SILAC media depends on two parameters: the labeling efficiency of proteins and the quantity of cells needed to form a sufficient number of cell-to-cell conjugates. The labeling efficiency for each cell type is evaluated after five serial cell passages and should exceed 95% labeling. If the labeling efficiency is not high enough, the cells should be cultured over an extended period of time until the threshold of 95% is reached.

To optimize cell culture, the size of cell culture flasks is adjusted to the number of cells necessary for coculture.

1. NK-92MI cell culture.
  a. NK-92MI are cultured in 30 mL RPMI SILAC medium in 75 cm^2^ flasks and used after 5 passages if the labeling efficiency of the cells exceeds 95%.
    ***Note:*** add the isotope-labeled amino acids freshly.
  b. Every 2–3 days, cells are counted to adjust the concentration at 3∗10^5^ cells/mL (1 passage).
  c. To proceed with step 2: 10^5^–10^6^ cells are sufficient for a preliminary test.
  d. To proceed with step 3: 3∗10^6^ are sufficient.
  e. To proceed with step 4: 35∗10^6^ NK-92MI cells are required.
2. MDA-MB-231 cell culture.
  a. MDA-MB-231 are cultured in 20 mL DMEM SILAC medium in 175 cm^2^ flasks and used after 7 passages.
    ***Note:*** add the isotope-labeled amino acids freshly.
  b. At the confluence, cells are washed with PBS to remove the excess medium.
  c. Add Trypsin-EDTA for 2 min.
  d. Collect the detached cells by adding MDA-MB-231 SILAC-cell culture medium.
  e. Seed the cells at a 1/5 dilution (1 passage).
  f. To proceed with step 2: 10^5^–10^6^ cells are sufficient for a preliminary test.
  g. To proceed with step 3: 10^6^ cells are sufficient.
  h. To proceed with step 4: 15∗10^6^ MDA-MB-231 cells are required.
    **CRITICAL:** It is necessary to perform a labeling efficiency test on heavy K and R to confirm that >95% of the proteins are heavy-labeled before proceeding to the next step. In case the labeling efficiency is < 95% go to problem 1.
    **CRITICAL:** It is necessary to cultivate both cell lines in SILAC media, even if light amino acids are used, to ensure that no excess amino acids are carried over into the next steps.

#### Determination of the labeling efficiency

**Timing: 2 days (for steps 3 to 22)**

In this section, the degree of incorporation of the isotope-labeled amino acids is determined by a mass spectrometric measurement.

3. Take an aliquot of the heavy labeled cells (cell culture section). A total of 10^5^–10^6^ cells are sufficient for the preliminary test.
4. Lyse cells with 100 μL of lysis buffer on ice.

***Note:*** prepare Lysis buffer freshly before use.

5. Vortex for 10 s at maximum setting.
6. Incubate cells at 4°C for 15 min to allow the mixture to sit.
7. Vortex for 10 s at maximum setting.
8. Centrifuge in a tabletop bench centrifuge at 4°C at maximum speed (16,000 g) to remove cell debris.
9. Transfer supernatant to a 2 mL Eppendorf tube.
10. Estimate protein concentration with BCA assay. The protein concentration should be between 0.5–2 mg/mL.
11. Add 5 μL of reduction buffer and incubate for 30 min at RT.
12. Add 5 μL of alkylation buffer and incubate for 30 min at RT in the dark.
13. Dilute sample 1:4 with dilution buffer to decrease urea concentration below 2 M.
14. Add trypsin buffer (substrate ratio of 1:50) for overnight at RT.
15. Add 10 μL of stop buffer.
16. Perform step 5 subsection, “digest clean up” to clean the peptides.
17. Reconstitute the samples in ST buffer B to obtain approximately 1 μg/μL.
18. Inject 1 μg of material into the LC-MS/MS with the parameters described in *the mass spectrometry resource parameters section.*
19. Analyze the data with MaxQuant following the protocol described in section: *automated interpretation of the mass spectra using MaxQuant*, with exceptions:
20. Under “variable modifications”, add Pro6 (determine arginine-to-proline conversion).
21. Select multiplicity 2 and mark the labels as Lys8 and Arg10. Do not select the “re-quantify” option (described in^10^).
22. The labeling efficiency should be calculated based on the non-normalized SILAC ratios and calculated for K and R separately.

***Note:*** Determine the incorporation rate as:

$$labeling\;efficency=1-\frac{1}{average\;ratio}$$

Complete labeling is considered when the incorporation rate is higher than 95%. Heavy proline (a side-product of arginine labeling) should not exceed 1%.

#### Cell-to-cell conjugation efficiency test

**Timing: 3 h (for step 23)**

The following section describes the formation and subsequent chemical cross-linking of cell-to-cell conjugates. For the isolation of the conjugates, natural killer cells and target cells are marked by two different fluorescent labels. Labeling can be achieved using immunofluorescence staining or expression of recombinant fluorescent proteins. Here, NK-92MI cells are labeled using a PE-Cy7 conjugated anti-CD56 antibody prior to conjugate formation, while MDA-MB-231 cells are identified by mEmerald-actin-fusion.^4^ Both cell types are co-incubated for 10 min to allow the formation of cell-to-cell conjugates and fixed with paraformaldehyde (PFA). Double-labeled conjugates are identified and isolated using FACS.

23. Calculate the number of cells required to reach the critical amount of cell-to-cell conjugates necessary for subsequent mass spectrometry analysis.

**Figure 2:**
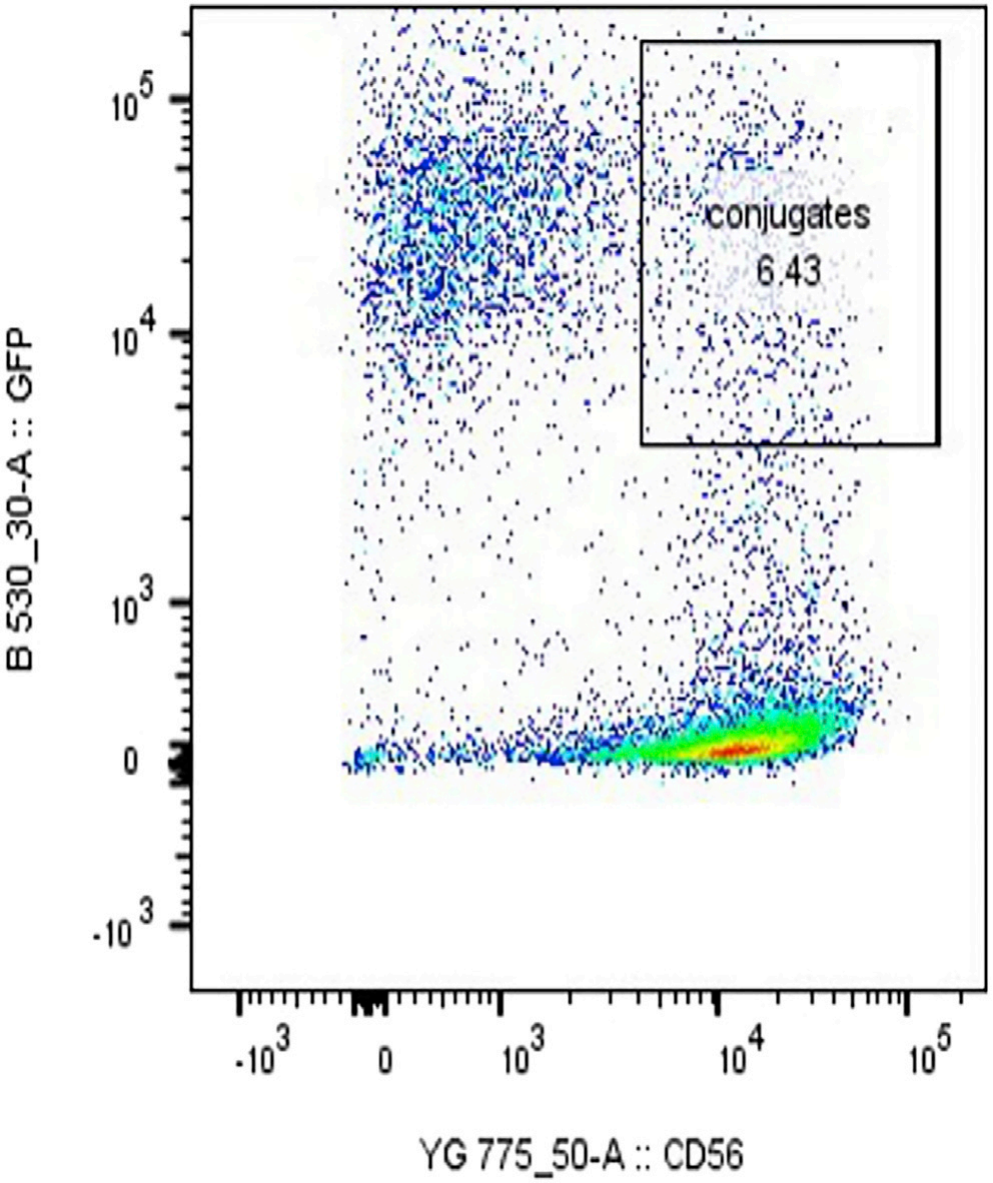
Example of dot plot of PE-Cy7 and mEmerald fluorescence intensity following NK92-MI cell-MDA-MB-231 cell conjugation formation Double positive (PE-Cy7+ mEmerald+) NK92-MI cell-MDA-MB-231 cell conjugates are selected. The evaluated percentage of cell-to-cell conjugates is 6,43%. Note: In our experimental conditions and using a ratio of NK92-MI cells:MDA-MB-231 cells of 3:1, we usually observed rate of 5%–10%.

***Note:*** An example is given below (Figure 2) following the method described in step 4 and step 5 with a 1:10 cell amount. In our experiment, we use the BD FACS Aria (Becton Dickinson Bioscience) for FACS analysis and sorting. Other instruments can be used, but the parameters have to be adjusted accordingly.

#### Conjugate formation and chemical cross-linking

**Timing: 3 h (for steps 24 to 28)**

A minimum of 25 μg of cell-to-cell conjugates (approximately 10^6^ conjugated cells) is necessary for mass spectrometry analysis. To calculate the number of NK-92MI cells and MDA-MB-231 cells that should be mixed in order to reach the critical number of cell-to-cell conjugates, both the NK-92MI:MDA-MB-231 cell ratio (i.e., 3:1) and the conjugation rate at this given ratio (i.e., 5%–10% of the total cell number) should be taken into account. It is important to test the cell-to-cell conjugation efficiency test first (conditions described in step 3) for further mass spectrometry experiments.

24. Staining of the NK-92 MI cells.
  a. Count NK-92MI cells using a Neubauer chamber with trypan blue solution (follow the manufacturer’s instructions).
  b. Transfer 35∗10^6^ NK-92MI cells in a 15 mL falcon tube.
  c. Centrifuge at 300 × *g* for 5 min.
  d. Discard the supernatant and resuspend the cells in 10 mL of AutoMACS Running Buffer.
  e. Centrifuge at 300 × *g* for 5 min.
  f. Discard the supernatant and resuspend the cells at 10∗10^6^ cells per mL.
  g. Distribute 1 mL per 15 mL falcon tube.
  h. Add 2.5 μL anti-human CD56-PECy7 antibody per million of cells and vortex briefly (2.5 μg/mL).
  i. Incubate at 4°C for 30 min in the dark.
  j. Add 10 mL of AutoMACS Running Buffer.
  k. Centrifuge at 300 × *g* for 5 min.
  l. Discard the supernatant and resuspend the cells at 9∗10^5^ cells in 200 μL of RPMI light medium.
  m. Incubate the cells for 15 min at 37°C in a cell culture incubator.

***Note:*** The required quantities should be adjusted according to the number of cells.

25. Detaching of the MDA-MB-231 cells.
  a. Remove the cell culture medium.
  b. Wash with 5 mL of DPBS.
  c. Add 3 mL of Trypsin-EDTA and incubate for 2 min at 37°C in a cell culture incubator.
  d. Stop trypsinization of the MDA-MB-231 cells by adding complete DMEM medium.
  e. Count MDA-MB-231 cells and resuspend at 3∗10^5^ cells in 200 μL in RPMI light culture medium.

***Note:*** The required quantities should be adjusted according to the number of cells.

26. NK-92MI cells and MDA-MB-231 cells co-culture, formation of the cell-to-cell conjugates.
  a. Conjugates and control cells.
    i. **Cell-to-cell Conjugates**: Mix 9∗10^5^ NK92-MI (200 μL) and 3∗10^5^ MDA-MB-231 (200 μL) in a round bottom tube (cell ratio 3:1). In total, 30∗10^6^ NK92-MI cells are conjugated with 10∗10^6^ MDA-MB-231 cells in 33 round bottom tubes.
    ii. **Control cells**: As a control, keep unmixed cells distributed in the same conditions as the mixed cells (200μL in round-bottom tubes).
    iii. Incubate 10 min at 37°C in a cell culture incubator.

***Note:*** In our example, 5∗10^6^ of MDA-MB-231 cells were distributed in 16 round bottom tubes at 3∗10^5^ cells per tube, and 5∗10^6^ of NK-92MI cells were distributed in 6 round bottom tubes at 9∗10^5^ cells per tube.

***Note:*** Excessive or ungentle centrifugation reduces the number of conjugated cells. See problem 2.

27. Stain for viability.
  a. Add 1 mL of PBS/Mg/Ca per tube.
  b. Centrifuge at 300 × *g* for 5 min.
  c. Discard the supernatant.
  d. Resuspend in PBS/Mg/Ca with 0.1× Live-or-Dye NucFix Red staining kit.
  e. Incubate for 15 min at 4°C.
  f. Add 1 mL of PBS/Mg/Ca per tube.
  g. Centrifuge at 300 × *g* for 5 min.

***Note:*** The required quantities should be recalculated according to the number of cells.

***Note:*** To reduce the risk of interfering with the subsequent steps, in particular mass spectrometric analysis, and recover unlabeled living cells, we recommend using (fixable) cell membrane impermeant viability dyes that specifically stain the nucleus of dead cells, such as Live-or-Dye NucFix™ Red.

***Note:*** Live-or-Dye NucFix Red must be diluted in PBS/Mg/Ca to keep conjugates maintained.

***Note:*** Resuspend by gently pipetting to avoid alteration of formed cell-to-cell conjugates.

28. Fix the cells.
  a. Add 200 μL of fixation buffer per tube.
  b. Incubate for 15 min at room temperature.
  c. Add 1 mL of PBS/Mg/Ca per tube.
  d. Centrifuge at 400 × *g* for 5 min.
  e. Add 200 μL PBS/Mg/Ca per tube.

***Note:*** PFA must be diluted in PBS/Mg/Ca to keep conjugates maintained.

***Note:*** The required quantities should be recalculated according to the number of cells.

#### Cell sorting

**Timing: 1 h (for step 29)**

**Timing: 1 day (for step 30)**

Step 5 describes the key steps for data acquisition and/or cell sorting. For further details on flow cytometry, the reader is referred to Cossarizza et al.^15^

**Figure 3:**
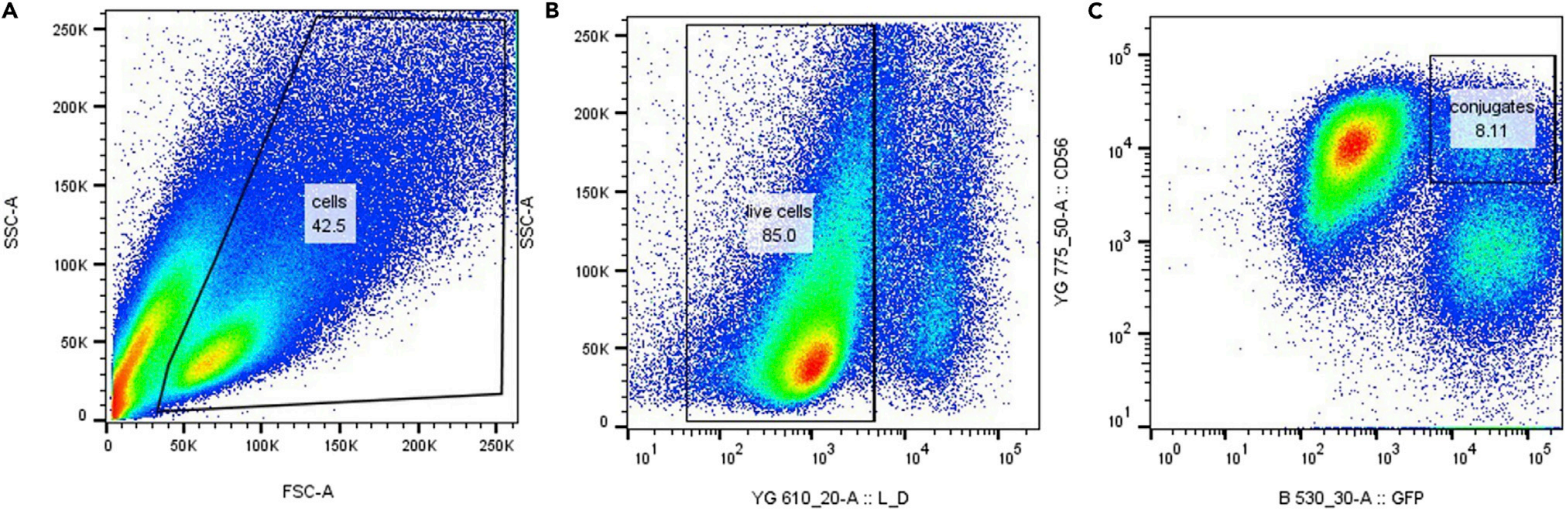
Gating strategy for identification and isolation of NK92-MI cell-MDA-MB-231 conjugates (A) Flow cytometry scatter FSC/SSC profile after co-incubation of effector and target cells. Cells are discriminated from cell debris based on size and granularity. The gate is intentionally extended (upper right corner) to maximize the number of cell-to-cell conjugates. (B) Live cells are discriminated from dead cells based on Live-or-Dye NucFix™ Red staining. (C) Double positive (PE-Cy7^+^ mEmerald^+^) NK92-MI -MDA-MB-231 cell conjugates are selected and sorted for subsequent mass spectrometry analysis.

29. FACS experiment set up.
  a. Create a new experiment.
  b. Design the gating strategy as illustrated in Figures 2, 3, and 4.

**Figure 4:**
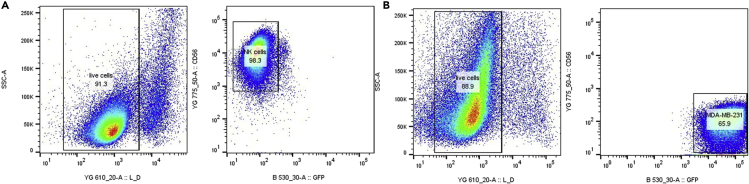
Gating strategy for identification and isolation of control cells (A) Live NK92-MI cells are discriminated from dead NK92-MI cells based on Live-or-Dye NucFix™ Red staining (left panel). PE-Cy7^+^ NK cells are selected and sorted for subsequent mass spectrometry analysis (right panel). (B) Live MDA-MB-231 cells are discriminated from dead MDA-MB-231 cells based on Live-or-Dye NucFix™ Red staining (left panel). mEmerald^+^ MDA-MB-231 cells are selected and sorted for subsequent mass spectrometry analysis (right panel).
  c. Use control cells to set appropriate PMT voltages.
  d. Proceed to data acquisition.
30. Isolation of control cells and cell-to-cell conjugates.
  a. Pipette gently to resuspend the cell suspension.
  b. Filter cell suspensions with 100μm cell strainers.
  c. Sort the Cell-to-Cell conjugates: sort NK92-MI cell-MDA-MB-231 cell conjugates by selecting the double positive PE-Cy7^+^ mEmerald^+^ cell population (Figure 3).
  d. Sort the Control cells: sort unmixed NK92-MI cells or unmixed MDA-MB-231 cells by selecting the single positive PE-Cy7^+^ cell population or single positive mEmerald^+^ cell population, respectively (Figure 4).
  e. Centrifuge at 500 × *g* for 10 min.
  f. Remove carefully as much supernatant as possible by pipette aspiration.

**Pause point:** The pellets can be stored at -80°C.

***Note:*** Recover at least 0.35 × 10^6^ cells (cell-to-cell conjugates or control cells) from cell sorting.

***Note:*** Pipet gently when resuspending cells to avoid dissociating cell-to-cell conjugates. If the sorting efficiency is below 30%, see problem 3.

**CAUTION**: This strategy allows selecting only NK92-MI cell-MDA-MB-231 cell conjugates, but the number of NK92-MI cells or MDA-MB-231 cells in the conjugate is uncertain. Several MDA-MB-231 and NK92-MI could be conjugated in the same cell complex. Filtering the cell suspension before the sorting will avoid larger aggregates of several cells.

### Production of protein extracts and mass spectrometry analysis

#### Protein extraction, digestion, and cleaning

**Timing: 2 days (for steps 31 to 34)**

Proteins are separated on a pre-casted SDS gel into three fractions to decrease the complexity of the sample. A lower complexity will increase the number of identified phosphopeptides. The SDS-gel functions two-fold: first, the heating of the samples reverses the PFA cross-links, and second, it removes substances that are interfering with the mass spectrometric analysis. Proteins are digested into peptides in the SDS-gel, and the resulting peptides are extracted. The contained phospho-peptides are enriched using metallo-chelate chromatography before being analyzed by mass spectrometry.

***Note:*** The starting material should not be below 0.35∗10^6^ cell-to-cell conjugates, which approximately corresponds to 25 μg per sample.

31. SDS-Page setup.
  a. Place the Bis-Tris gel in the tank.
  b. Fill the tank with running buffer.
32. Sample cleaning and fractionation by SDS-PAGE.
  a. Resuspend the PFA-treated samples in 25 μL 2× Laemmli buffer and add 5 μL 10% SDS.
  b. Vortex vigorous for 20 s.
  c. Incubate 10 min at 95°C.
  d. Put the samples on ice for 5 min.
  e. Centrifuge for 10 min at maximum speed in a tabletop centrifuge (16,000 × *g*).
  f. Load the samples and protein size standard in the different wells.
  g. Program the power supply to 120 V constant per gel.
  h. Run the gel until the running front reaches approximately half of the gel.
  i. Stain the gel with Coomassie brilliant blue staining solution.
  j. Cut each lane with a razor blade in three equal slices. Every slice should be cut into cubes of approximately 1 mm^2^ size to optimize further cleaning and increase penetration of the different reagents.
  k. Place all cubes from every slice in a different 2 mL Eppendorf tube.

***Note:*** Proteins should be clearly fractionated into different bands. See problem 4.

***Note:*** The whole sample should run through the gel with no remaining sample at the top of the well. See problem 5.

33. Protein digestion.
  a. Wash the gel pieces with 400 μL of washing buffer A for 10 min in a thermomixer, 800 g, at RT.
  b. Discard the supernatant.
  c. Wash the gel pieces with 400 μL of washing buffer B for 10 min in a thermomixer, 800 × *g*, at RT.
  d. Discard the supernatant.
  e. Repeat the steps (33a to 33c) four times.
  f. After discarding the supernatant, incubate the sample with 200 μL of reduction buffer for 20 min in a thermomixer at 800 × *g*.
  g. Discard the supernatant.
  h. Add 200 μL of alkylation buffer for 30 min in a thermomixer at 800 × *g* (in the dark) at RT.
  i. Discard the supernatant.
  j. Add 200 μL washing buffer B for 10 min in a thermomixer at 800 × *g*.
  k. Discard the supernatant.
  l. Add 150 μL digestion buffer.
  m. Incubate the samples for 16 h at 37°C.
  n. Stop the digestion by adding 15 μL of stop buffer (final concentration 0.1% formic acid).
  o. Place the supernatant in a fresh 2 mL Eppendorf tube.
  p. Add 150 μL of ST buffer C to the gel pieces and incubate the sample for 30 min at 800 g at RT.
  q. Pool the supernatant from the gel pieces with the previous extraction in the Eppendorf tubes.
  r. Dry the samples in a speed-vac for 2 h at 45°C.

**CAUTION:** The coomassie brilliant blue dye from the gel pieces should vanish for optimal digestion. If the coomassie brilliant blue dye remains, add 3 more washes (step 33c).

**CAUTION**: The gel pieces should be wet in all the procedures; otherwise, the reproducibility of digestion will be compromised.

**CRITICAL:** If the gel pieces absorb all the buffer, add 50 μL digestion buffer. In case the digestion buffer is increased, the stop buffer should also be adjusted to a final concentration of 0.1% formic acid.

**Pause point:** The dried peptides can be stored for 1 week at -20°C.

34. Digest clean-up.
  a. Acidify digest (∼50 μL) by adding ST buffer A (check pH < 3).
  b. Punch out 2 discs of Octadecyl (C18)-bonded silica material with a flat tip gauge 16 needle.
  c. Push the 2 discs into a GPS200 pipette tip using PEEK tubing.
  d. Add 20 μL ST buffer A to the disc.
  e. Centrifuge the tip at 1,000 × *g* for 1 min.
  f. Add 20μL ST buffer C to the disc.
  g. Centrifuge the tip at 1,000 × *g* for 1 min.
  h. Add 20 μL ST buffer B to the disc.
  i. Centrifuge the tip at 1,000 × *g* for 1 min.
  j. Repeat steps 34f to 34i twice.
  k. Load acidified sample into the tip.
  l. Centrifuge the tip at 1,000 × *g* for 3 min.
  m. Wash the sample that is retained in the tip with 20 μL ST buffer B.
  n. Centrifuge the tip at 1,000 × *g* for 1 min.
  o. Repeat steps 34m and 34n 3 times.
  p. Elute bound peptides with 50 μL of ST buffer C.
  q. Transfer eluate in measuring plate.
  r. Snap freeze and lyophilize in speed vac.

**Pause point:** The purified peptides can be stored one month at -80°C.

#### Phosphopeptide enrichment

**Timing: 1 h (for steps 35 to 36)**

Phosphopeptides are usually low abundant and are difficult to be detected in the mass spectrometer. Therefore, the phosphopeptides need to be enriched using a specific metallo-chelate chromatography.

35. Reconstitute the samples in the starting buffer and perform the phosphopeptide enrichment as specified in High-Select Fe-NTA Phosphopeptide Enrichment Kit.

***Note:*** this step enables the enrichment of phosphorylated peptides from protein extracts using iron-chelate resin in spin columns. The capacity of the columns ranges from 0.5 to 5 mg of total digested protein sample and can enrich up to 150 μg phosphopeptides with high selectivity.

36. In the last step of the kit protocol, resuspend the material in 12 μL of 0.1% formic acid to inject into the mass spectrometer.

**CRITICAL:** check the color of the Fe^3+^-NTA beads. The color should be black. If the beads turn yellow, the beads are oxidized and will not work.

***Note:*** if the analysis of the sample does not show enough phosphopeptides, See the troubleshooting of the High-Select Fe-NTA Phosphopeptide Enrichment Kit.

**Pause point:** The eluted peptides can be stored at -80°C for one month.

#### Mass spectrometry analysis (MS)

**Timing: 1 day (dependent on the computer hardware available) (for step 37)**

The recorded mass spectrometric data has to be matched to peptides and proteins. For this, we use the MaxQuant software package. MaxQuant demands significant computing resources. In our case, we used a 72-core computer to analyze the data. Using a smaller computer will prolong the time needed for the analysis.

37. Inject 0.2–0.5 μg of material into an LC-MS/MS. It is very important to inject exactly the same amount in all the samples (peptide abundance measured by Pierce™ BCA Protein Assay Kit).

***Note:*** the parameters that are described below are a guide for a specific mass spectrometer, but others could be used to perform MS analysis.

***Optional:*** The amount of phosphopeptides to inject could differ depending on the peptide abundance, the mass spectrometer, and the nanoHPLC system. Those conditions have to be adjusted injecting the corresponding volume into LC-MS/MS. For a Q Exactive HF (Thermo Scientific) 0.1 μg of material is enough to generate a robust dataset (around 2,500 peptides per measurement, which corresponds to approximately 2 × 10^9^ of maximum total ion count (TIC)).

To interpret the mass spectrometry data, use the MaxQuant software package (here, we use version 1.6.17.0) with the following search parameters. The rest of the MaxQuant parameters should be maintained as default for optimal data processing.^12^^,^^16^^,^^17^

**Table:** 

Parameter	Value
Version	2.0.1.0
Include contaminants	TRUE
PSM FDR	0.01
PSM FDR Crosslink	0.01
Protein FDR	0.01
Site FDR	0.01
Use Normalized Ratios For Occupancy	TRUE
Min. peptide Length	7
Min. score for unmodified peptides	0
Min. score for modified peptides	40
Min. delta score for unmodified peptides	0
Min. delta score for modified peptides	6
Min. unique peptides	0
Min. razor peptides	1
Min. peptides	1
Fixed modification	
Use only unmodified peptides and	TRUE
Modifications included in protein quantification	Oxidation (M);Acetyl (Protein N-term)
Peptides used for protein quantification	Razor
Discard unmodified counterpart peptides	TRUE
Label min. ratio count	2
Use delta score	FALSE
iBAQ	FALSE
iBAQ log fit	FALSE
Match between runs	TRUE
Matching time window [min]	0.7
Match ion mobility window [indices]	0.05
Alignment time window [min]	20
Alignment ion mobility window [indices]	1
Find dependent peptides	TRUE
Dependent peptide FDR	0.01
Mass bin size	0.0065
Decoy mode	revert
Include contaminants	TRUE
Advanced ratios	TRUE
Second peptides	TRUE
Stabilize large LFQ ratios	TRUE
Separate LFQ in parameter groups	FALSE
Require MS/MS for LFQ comparisons	TRUE
Calculate peak properties	FALSE
Main search max. combinations	200
Advanced site intensities	TRUE
Write msScans table	FALSE
Write msmsScans table	TRUE
Write ms3Scans table	TRUE
Write allPeptides table	TRUE
Write mzRange table	TRUE
Write DIA fragments table	FALSE
Write DIA fragments quant table	FALSE
Write pasefMsmsScans table	TRUE
Write accumulatedMsmsScans table	TRUE
Max. peptide mass [Da]	4600
Min. peptide length for unspecific search	8
Max. peptide length for unspecific search	25
Razor protein FDR	TRUE
Disable MD5	FALSE
Max mods in site table	3
Match unidentified features	FALSE
Evaluate variant peptides separately	TRUE
Variation mode	None
MS/MS tol. (FTMS)	20 ppm
Top MS/MS peaks per Da interval. (FTMS)	12
Da interval. (FTMS)	100
MS/MS deisotoping (FTMS)	TRUE
MS/MS deisotoping tolerance (FTMS)	7
MS/MS deisotoping tolerance unit (FTMS)	ppm
MS/MS higher charges (FTMS)	TRUE
MS/MS water loss (FTMS)	TRUE
MS/MS ammonia loss (FTMS)	TRUE
MS/MS dependent losses (FTMS)	TRUE
MS/MS recalibration (FTMS)	FALSE
MS/MS tol. (Unknown)	20 ppm
Top MS/MS peaks per Da interval. (Unknown)	12
Da interval. (Unknown)	100
MS/MS deisotoping (Unknown)	TRUE
MS/MS deisotoping tolerance (Unknown)	7
MS/MS deisotoping tolerance unit (Unknown)	ppm
MS/MS higher charges (Unknown)	TRUE
MS/MS water loss (Unknown)	TRUE
MS/MS ammonia loss (Unknown)	TRUE
MS/MS dependent losses (Unknown)	TRUE
MS/MS recalibration (Unknown)	FALSE
Site tables	Deamidation (NQ)Sites.txt;Oxidation (M)Sites.txt;Phospho (STY)Sites.txt
Enzyme	Trypsin/P
Enzyme mode	Specific
Use enzyme first search	FALSE
Use variable modifications first search	FALSE
Requantify	TRUE
Multiplicity	2
Max. missed cleavages	2
Max. labeled AAs	3
Labels0	
Labels1	Arg10;Lys8
LC-MS run type	Standard

***Note:*** The MaxQuant software has high demands on computer hardware. The runs may take a long time on a normal computer.

#### Bioinformatic analysis and data interpretation

**Timing: 2 days (for steps 38 to 46)**

**Figure 5:**
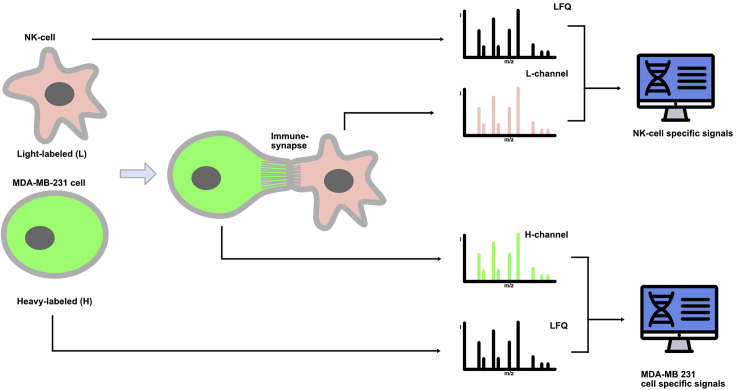
Data analysis scheme Cell types are SILAC labeled. Both the single cells and the cell-to-cell conjugates are measured by mass spectrometry. The two different SILAC isotope markers are used as different channels and compared to the single cells using LFQ quantification to identify cell type-specific events.

In this step, the data is analyzed using statistical tools (Figure 5).

The MaxQuant search engine generates several files while processing the raw data from ThermoFisher instruments. To extract the files needed for the analysis: go to the combined folder, and then to the txt folder. The samples are analyzed by R-based programming or Perseus.^18^^,^^19^ Perseus is convenient for filtering data (114–116). For the quantification (117–121) we recommend R-based scripting to analyze the data. An example of the data analysis is shown in Table S1.

38. Filter out the phosphopeptides belonging to proteins that are contaminants, reverse or identified by site in proteinGroups.txt file and Phospho (STY)Sites.txt file.
39. In Phospho (STY)Sites.txt file, Phosphopeptides with at least two individual SILAC ratios in each type of sample can be selected with the following threshold parameters:
  a. score > 40.
  b. score for localization > 40.
  c. PEP <0.015.
40. Separate the filtered phosphopeptides by the two SILAC channels. In this case, the Light (L) channel corresponds to Natural Killer cells (NK92-MI), and the Heavy channel (H) corresponds to MDA-MB-231 cells.

***Note:*** a minimum of two biological replicates have to be considered to calculate the abundance of every single phosphosite.

41. The phosphosites are quantified using the Intensity that is differentially labeled by SILAC. To validate a significant change of phosphorylation, compare the Intensity H from the MDA-MB-231 cells (Intensity H MDA-MB-231) to Intensity H from the conjugates (Intensity H MDA-MB-231 - NK92-MI), or the Intensity L from NK92-MI cells (Intensity L NK92-MI) to the Intensity L from the conjugate (Intensity L MDA-MB-231 - NK92-MI).
42. **ONLY** differences of the absolute value of

$$\left|\log_{10}\frac{Intensity\;H\;MDA-NK}{Intensity\;H\;MDA}\right|>5$$

are considered as valid phosphorylation (positive) or dephosphorylation (negative). The rest of the phosphosites will be discarded as the signal is not sufficient to distinguish from background signals.

43. The abundance changes in the phosphosites also depend on the differences in protein abundance between those two samples. We monitor this difference by Z-scoring of the LFQ Intensity H in proteingroups.txt file.
44. $\left|\log_{2}\frac{LFQ\;Intensity\;H\;MDA-NK}{LFQ\;Intensity\;H\;MDA}\right|<1$ will not be considered as protein abundance change.
45. $\left|\log_{2}\frac{LFQ\;Intensity\;H\;MDA-NK}{LFQ\;Intensity\;H\;MDA}\right|>1$ Proteins that include phosphosites changing in the same direction will be discarded.
46. The SILAC label compares the protein abundance between MDA-MB-231 and NK92-MI in conjugates.

***Note:*** the SILAC pairs in NK92-MI will be considered as an artifact (only L label in the sample), but the SILAC pair in MDAs (H label) will be considered to perform a quality control for labeling efficiency.

## Expected outcomes

The provided datasets originate from an analysis using the described method. The data analysis of the dataset leads to the identification of 1197 phosphoproteins and 2800 phosphopeptides, which can be assigned to different cell types of cell-to-cell conjugates. Experiments using other cell types should identify phosphoproteins and -peptides in the same range.

## Limitations

The analysis is based on SILAC-labeling to distinguish where the phosphorylated peptides originate. Thus, the method can only be used on cells that can be SILAC-labeled. The number of cell-to-cell conjugates formed by the two cell populations can vary depending on the cell type and might be a limiting factor for some cell type combinations. If one cell type can form homotypic cell-to-cell conjugates, the strategy for FACS needs to be adjusted and fitted to select conjugated cells from single cells. A minimal number of phosphorylated peptides are necessary for the mass spectrometric analysis to get sufficient identifications. Depending on the conjugation efficiency, the amounts have to be adjusted. In general, more replicates will lead to a more robust coverage of the phospho-proteome.

## Troubleshooting

### Problem 1

The labeling efficiency of the cells is <95% due to problems with the dialyzed serum or the number of passages is not enough to incorporate all the labeled amino acids (steps 1 and 2).

### Potential solution

Wash cells with PBS and perform 4 more passages with a fresh dialyzed serum before a new test. Check if the SILAC media does contain the right amount of SILAC amino acids for labeling.

### Problem 2

Centrifugation and pipetting before the fixation step could alter cell-to-cell conjugates (steps 26–28). The sheer forces within the pipette can separate cells from each other.

### Potential solution

Pipet gently and limit washing steps by pre-labeling cells for viability before NK92-MI cells and MDA-MB-231 conjugation. The 10 min co-culture time is insufficient to induce strong NK92-MI cell cytotoxic activity, specifically with MDA-MB-231 characterized by their resistance to NK92-MI cell attack.

### Problem 3

The sorting efficiency (for both NK92-MI cell-MDA-MB-231 cell conjugates and unmixed cells), as defined by the number of positive events sorted divided by the number of positive events detected, has to be larger than 30%.

### Potential solution

The sorting efficiency depends on several parameters that could be modified to improve it:

• Aggregates formed by cell clustering can be limited by a filtration step with 100 cell strainers before the sorting.
• The cell concentration per mL is important to limit the exclusion of positive events because of too close proximity to a negative event. The cell suspension could be diluted to limit this phenomenon.
• A first sorting can improve the percentage of positive events in the cell suspension based on yield and not purity. The machine’s settings on yield mode allow the recovery of all positive events, despite the proximity to negative events that could be included in the sorted drop. The recovered cell suspension is enriched in positive events and can be sorted again on purity mode. The machine will exclude all drops that could contain any non-target events.

### Problem 4

There is no clean separation between the different bands in the SDS-PAGE.

### Potential solution

Generally, this is due to the high concentration of PFA or high amounts of DNA in the sample. Add 8 μL of 10% SDS instead of 5 μL and boil for 15 min at 95°C. Use sonication to break down the DNA or add 5 μL of Benzonase for 20 min at RT.

### Problem 5

The sample is not homogeneous, and some of the samples is not passing through the gel or is trapped in the gel pocket.

### Potential solution

The sample was not soluble because of the cell debris. Perform an ultra-centrifugation at 100,000 g for 20 min after boiling the sample, recovering the non-solid phase (upper phase) before loading it onto the SDS-PAGE.

## Resource availability

### Lead contact

Further information and requests for resources and reagents should be directed to and will be fulfilled by the lead contact, Gunnar Dittmar (gunnar.dittmar@lih.lu).

### Materials availability

This study did not generate new unique reagents.

## Data Availability

The mass spectrometric dataset generated during this study are available at MASSIVE proteomeXchange: MSV000090645. ftp://massive.ucsd.edu/MSV000090645/

## Supplemental information

Table S1. Test dataset

## References

[1] Dustin M.L. (2014). The immunological synapse. Cancer Immunol. Res..

[2] Orange J.S. (2008). Formation and function of the lytic NK-cell immunological synapse. Nat. Rev. Immunol..

[3] Wurzer H., Hoffmann C., Al Absi A., Thomas C. (2019). Actin cytoskeleton straddling the immunological synapse between cytotoxic lymphocytes and cancer cells. Cells.

[4] Al Absi A., Wurzer H., Guerin C., Hoffmann C., Moreau F., Mao X., Brown-Clay J., Petrolli R., Casellas C.P., Dieterle M. (2018). Actin cytoskeleton remodeling drives breast cancer cell escape from natural killer-mediated cytotoxicity. Cancer Res..

[5] Biolato A.M., Filali L., Wurzer H., Hoffmann C., Gargiulo E., Valitutti S., Thomas C. (2020). Actin remodeling and vesicular trafficking at the tumor cell side of the immunological synapse direct evasion from cytotoxic lymphocytes. Int. Rev. Cell Mol. Biol..

[6] Wurzer H., Filali L., Hoffmann C., Krecke M., Biolato A.M., Mastio J., De Wilde S., François J.H., Largeot A., Berchem G. (2021). Intrinsic resistance of chronic lymphocytic leukemia cells to NK cell-mediated lysis can Be overcome in vitro by pharmacological inhibition of Cdc42-induced actin cytoskeleton remodeling. Front. Immunol..

[7] Diella F., Haslam N., Chica C., Budd A., Michael S., Brown N.P., Trave G., Gibson T.J. (2008). Understanding eukaryotic linear motifs and their role in cell signaling and regulation. Front. Biosci..

[8] Friedl P., den Boer A.T., Gunzer M. (2005). Tuning immune responses: diversity and adaptation of the immunological synapse. Nat. Rev. Immunol..

[9] Lanzavecchia A. (1985). Antigen-specific interaction between T and B cells. Nature.

[10] Ong S.-E., Blagoev B., Kratchmarova I., Kristensen D.B., Steen H., Pandey A., Mann M. (2002). Stable isotope labeling by amino acids in cell culture, SILAC, as a simple and accurate approach to expression proteomics. Mol. Cell. Proteomics.

[11] Deng J., Erdjument-Bromage H., Neubert T.A. (2019). Quantitative comparison of proteomes using SILAC. Curr. Protoc. Protein Sci..

[12] Cox J., Mann M. (2008). MaxQuant enables high peptide identification rates, individualized p.p.b.-range mass accuracies and proteome-wide protein quantification. Nat. Biotechnol..

[13] Tyanova S., Mann M., Cox J. (2014). MaxQuant for in-depth analysis of large SILAC datasets. Methods Mol. Biol..

[14] Ong S.E., Foster L.J., Mann M. (2003). Mass spectrometric-based approaches in quantitative proteomics. Methods.

[15] Cossarizza A., Chang H., Radbruch A., Acs A., Adam D., Adam-Klages S., Agace W.W., Aghaeepour N., Akdis M., Allez M. (2019). Guidelines for the use of flow cytometry and cell sorting in immunological studies (second edition). Eur. J. Immunol..

[16] Cox J., Matic I., Hilger M., Nagaraj N., Selbach M., Olsen J.V., Mann M. (2009). A practical guide to the MaxQuant computational platform for SILAC-based quantitative proteomics. Nat. Protoc..

[17] Tyanova S., Temu T., Cox J. (2016). The MaxQuant computational platform for mass spectrometry-based shotgun proteomics. Nat. Protoc..

[18] Tyanova S., Temu T., Sinitcyn P., Carlson A., Hein M.Y., Geiger T., Mann M., Cox J. (2016). The Perseus computational platform for comprehensive analysis of (prote)omics data. Nat. Methods.

[19] Tyanova S., Cox J., von Stechow L. (2018). Cancer Systems Biology Methods in Molecular Biology.

